# Butanol production under microaerobic conditions with a symbiotic system of *Clostridiumacetobutylicum* and *Bacillus cereus*

**DOI:** 10.1186/s12934-016-0412-z

**Published:** 2016-01-14

**Authors:** Pengfei Wu, Genyu Wang, Gehua Wang, Børre Tore Børresen, Hongjuan Liu, Jianan Zhang

**Affiliations:** Institute of Nuclear and New Energy Technology, Tsinghua University, Beijing, 100084 People’s Republic of China; Statoil Petroleum AS, 4035 Stavanger, Norway

**Keywords:** ABE fermentation, Symbiotic system, *Clostridium acetobutylicum* TSH1, *Bacillus cereus* TSH2, Oxygen

## Abstract

**Background:**

One major problem of ABE (acetone, butanol and ethanol) fermentation is high oxygen sensitivity of *Clostridium acetobutylicum*. Currently, no single strain has been isolated or genetically engineered to produce butanol effectively under aerobic conditions. In our previous work, a symbiotic system TSH06 has been developed successfully by our group, and two strains, *C. acetobutylicum* TSH1 and *Bacillus cereus* TSH2, were isolated from TSH06.

**Results:**

Compared with single culture, TSH06 showed promotion on cell growth and solvent accumulation under microaerobic conditions. To simulate TSH06, a new symbiotic system was successfully re-constructed by adding living cells of *B. cereus* TSH2 into *C. acetobutylicum* TSH1 cultures. During the fermentation process, the function of *B. cereus* TSH2 was found to deplete oxygen and provide anaerobic environment for *C. acetobutylicum* TSH1. Furthermore, inoculation ratio of *C. acetobutylicum* TSH1 and *B. cereus* TSH2 affected butanol production. In a batch fermentation with optimized inoculation ratio of 5 % *C. acetobutylicum* TSH1 and 0.5 % *B. cereus* TSH2, 11.0 g/L butanol and 18.1 g/L ABE were produced under microaerobic static condition. In contrast to the single culture of *C. acetobutylicum* TSH1, the symbiotic system became more aerotolerant and was able to produce 11.2 g/L butanol in a 5 L bioreactor even with continuous 0.15 L/min air sparging. In addition, qPCR assay demonstrated that the abundance of *B. cereus* TSH2 increased quickly at first and then decreased sharply to lower than 1 %, whereas *C. acetobutylicum* TSH1 accounted for more than 99 % of the whole population in solventogenic phase.

**Conclusions:**

The characterization of a novel symbiotic system on butanol fermentation was studied. The new symbiotic system re-constructed by co-culture of *C. acetobutylicum* TSH1 and *B. cereus* TSH2 showed excellent performance on butanol production under microaerobic conditions. *B. cereus* TSH2 was a good partner for *C. acetobutylicum* TSH1 by providing an anaerobic environment. During fermentation process, the high ratio of *Clostridium* and low ratio of *Bacillus* composition indicated that this symbiotic system was an effective and easily controlled cultivation model for ABE fermentation under microaerobic conditions.

## Background

The endospore forming, gram-positive *Clostridium acetobutylicum* is a classic example of fermentative obligate anaerobes. Under anaerobic conditions, it ferments sugars or starch to acetate and butyrate and then shifts them to solvents such as butanol, acetone and ethanol. In these solvents, butanol has been regained attention in recent years as an attractive biofuel, because it exhibits superior performance in terms of energy content, blending ability, volatility, vapor pressure and corrosiveness [1, 2]. Thus, genetic engineering and bioprocess technologies are ranked highly among recent efforts to increase productivity and economic competitiveness of the fermentation route for butanol production [3–6]. Although many improvements and developments have been achieved over the past three decades,conventional ABE (acetone, butanol and ethanol) fermentation still has faced to a number of challenges [7]. One major problem is high oxygen sensitivity of *C. acetobutylicum*. O’Brien and Morris reported that glucose consumption rate decreased and cell growth was halted as well as DNA, RNA, protein syntheses were prevented if cells of *C. acetobutylicum* were exposed to high concentration of oxygen [8]. Compared with aerobes, the strict anaerobes need special equipment and complicated operation to eliminate oxygen in the culture medium, for example, adding reducing agents or flushing with N_2_ gas, which increased the total cost of ABE fermentation. Furthermore, aerobic metabolism could reach higher cell density without accumulating higher level of acids, and improve butanol productivity [9]. However, for batch fermentation, the productivity of butanol is often lower than 0.3 g/L h which decrease the economy of ABE fermentation [10]. So, high oxygen tolerance is believed to be one desirable behavior of *C. acetobutylicum*.

In nature, microorganisms often exist in highly diverse and complex communities rather than live in isolation. In these natural communities,microorganisms coexist stably by interacting with each other and exert various functions more effectively than single culture [11]. For example, natural cooperation were often observed in interspecies hydrogen transfer within methanogenic systems, and in the degradation of xenobiotics such as halogenated organic compounds [12]. As an obligate anaerobe, *Clostridiums* were not able to survive in aerobic environments, but were often detected with aerobic bacteria simultaneously in habitats which were exposed to oxygen environments such as rice paddy soils and water retting pond. In these habitats, aerobic bacteria were considered to be essential for *Clostridiums* by supplying an anaerobic environment and consuming harmful metabolites. The cooperation between *Clostridiums* and aerobic bacteria were observed not only in natural habitats but also in many artificial systems. For example, by controlling the volumetric transport rate of oxygen, an artificial symbiosis was established between *C. phytofermentans* and other two yeast species. In this system, both yeasts were capable of providing respiratory protection to *C. phytofermentans*, and the symbiosis was stable for almost 2 months under semi-aerobic conditions and produced more ethanol than any single cultures [11].

Co-culture of *Clostridium* and other bacteria which have enzymes capable of hydrolyzing cellulose and hemicellulose were induced in utilization of cellulosic biomass. In these co-culture systems, cellulose and hemicellulose were first hydrolyzed to glucose or butyric acid by cellulolytic strains, and then butanol was obtained subsequently by adding solvent-producing *Clostridium* species [13–16]. These researches were carried out under strict anaerobic conditions as all of the strains used were anaerobic bacteria. However, there were few reports on butanol fermentation by *Clostridium* and aerobic bacteria. Stevens used a co-culture of *C. beijerinkii* and *Bacillus cereus* to ferment cheese whey, and found that higher concentration of butanol was obtained in the mixed culture without sacrificing butyric acid [17]. In order to enhance solvent production from cassava starch, *B. subtilis* was co-cultured with *C. butylicum*. In this co-culture system, amylase activity was increased by tenfold and solvent production was enhanced by 6.5-fold compared to the pure culture of *Clostridium* [18]. Abd-Alla founded that co-culture of *C. acetobutylicum* and *B. subtilis* were able to use spoilage date palm fruits as a substrate to produce butanol without any anaerobic pretreatment [19]. In an artificial syntrophic co-culture system, *Bacillus* was founded to be a good partner for creating anaerobic environment and pre-saccharification of substrate for co-cultured *Clostridium* strain as it showed multiple extracellular enzyme activities including lipase, protease, a-amylase, pectinase and cellulose [20].

Though some reports confirmed that aerobic bacteria, such as *B. subtilis* and *B. cereus*, had a positive interaction with anaerobic *Clostridium*, the role of aerobic bacteria was not established clearly. Firstly, aerobic bacteria in the co-culture system were considered to sustain anaerobic environment for *Clostridiums*, however, there was lack of oxygen variation data during fermentation process in these literatures. Secondly, different micro-organisms in the co-culture system may compete for substrates, therefore, the product yield and productivity would be influenced compared with single cultures. Moreover, the complicated community interactions between the different microorganisms in co-culture systems were still unclear. So, further study would be necessary.

In our previous work, a stable symbiotic system TSH06 was developed and anaerobic *C. acetobutylicum* TSH1 and aerobic *B. cereus* TSH2 were isolated from TSH06 [21]. In this study, a new symbiotic system was successfully re-constructed by adding living cells of *B. cereus* TSH2 to *C. acetobutylicum* TSH1 culture, and some important characters of ABE fermentation by this symbiotic system, such as dissolved oxygen and inoculation ratio, were investigated. In addition, butanol production and abundance of each strain during the fermentation process were also discussed.

## Results and discussion

### Cell growth and solvent production with selected different strains under microaerobic conditions

**Fig. 1:**
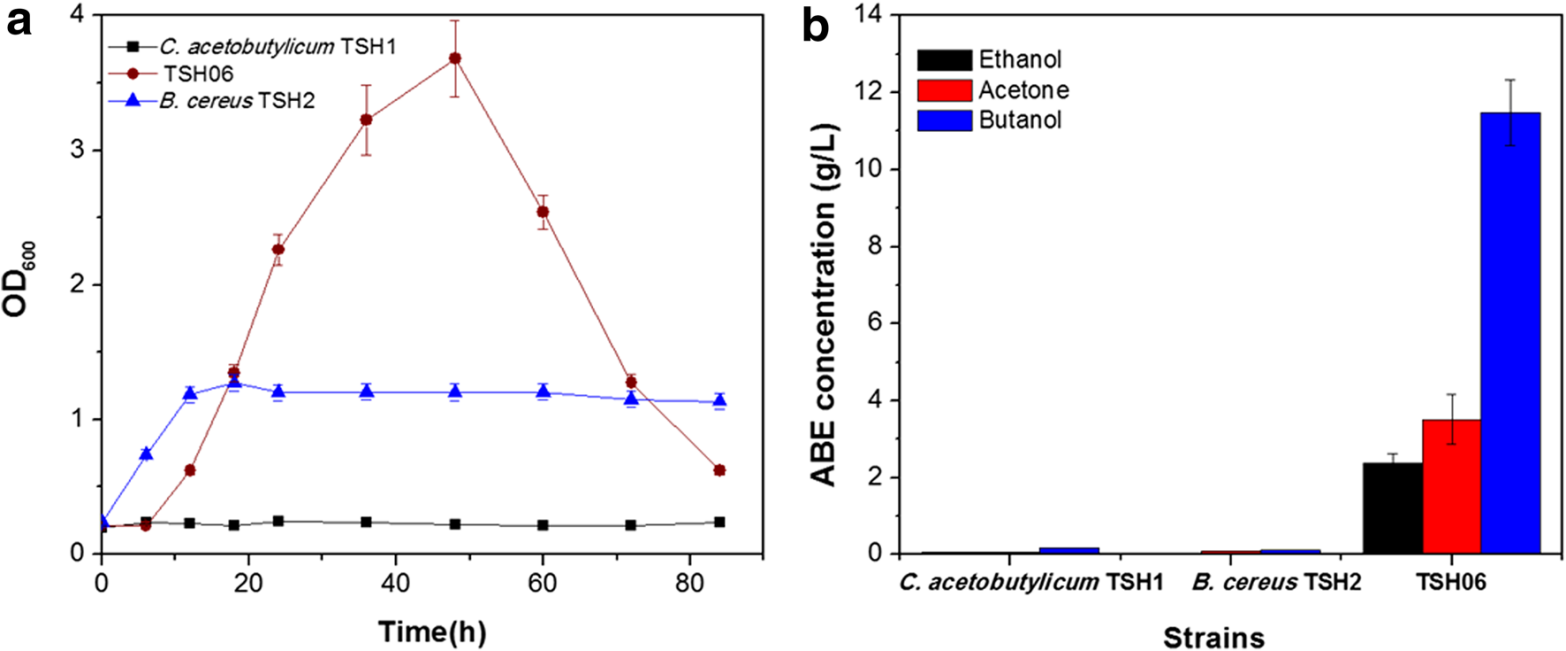
Cell growth and ABE solvents production by TSH06, *C. acetobutylicum* TSH1 and *B. cereus* TSH2. **a** Time course of cell growth. **b** Acetone, butanol and ethanol observed in final broth. The cultures were carried out in 100 mL shaken flasks with 60 mL P_2_ medium, and incubated statically at 37 °C without anaerobic treatment for 84 h

In general, symbiotic system or co-culture system appears to be advantageous over single culture because of the potential for cooperative utilization of the metabolic pathways of all involved organisms [22]. Cell growth of one micro-organism may be enhanced by activities of other micro-organisms. Figure 1a shows the cell growth of symbiotic system TSH06, *C. acetobutylicum* TSH1 and *B. cereus* TSH2 under microaerobic static conditions, respectively. Like most of *Clostridium*s, *C. acetobutylicum* TSH1 cannot grow in P_2_ medium under microaerobic conditions, and OD_600_ was still below 0.3 after 30 h. Different from *C. acetobutylicum* TSH1, *B. cereus* TSH2 survived with a low biomass in P_2_ medium, OD_600_ reached 1.2 at 12 h and then remained steady. Both of *C. acetobutylicum* TSH1 and *B. cereus* TSH2 displayed poor growth when they were cultured individually, however, promotion of cell growth occurred in symbiotic system TSH06. At the initial 12 h, cell growth rate of TSH06 was comparable with that of *B. cereus* TSH2. After that, OD_600_ of TSH06 increased quickly and reached 3.7 at 48 h, while OD_600_ of *B. cereus* TSH2 remained at 1.2. After 48 h, OD_600_ of TSH06 decreased owing to the butanol toxicity [5].

In symbiotic system TSH06,not only cell growth but also solvent producing ability was enhanced. Figure 1b shows solvent production in ABE fermentation under microaerobic conditions. With TSH06, 11.5 g/L butanol, 3.5 g/L acetone, and 2.4 g/L ethanol were obtained. However, ABE couldn’t be produced in single culture of *C. acetobutylicum* TSH1 or *B. cereus* TSH2 respectively. These results demonstrated that the symbiotic system had potential advantage in ABE fermentation, as it didn’t need any anaerobic pretreatment, such as reducing agent addition or N_2_ flushing. That made ABE fermentation more economical and easier to be operated.

### Re-construction of symbiotic system by adding various *B. cereus* TSH2 cultures into *C. acetobutylicum* TSH1

In symbiotic system TSH06, there were only two micro-organisms, *C. acetobutylicum* TSH1 and *B. cereus* TSH2. *C. acetobutylicum* TSH1 was an anaerobic, solvent-producing strain, and *B. cereus* TSH2 was an aerobic, non-solvent-producing strain. Two positive interactions may exist in TSH06. One interaction is *B. cereus* TSH2 protecting *C. acetobutylicum* TSH1 from environmental influences, such as oxygen toxicity; another is the products of *B. cereus* TSH2 supplying *C. acetobutylicum* TSH1 as substrate. To explore the interactions mentioned above, symbiotic systems were re-constructed by adding various components of *B. cereus* TSH2 cultures into *C. acetobutylicum* TSH1 culture. *B. cereus* TSH2 cultures included cells, cell-free cultures, cell-free enzymes, sterilized cultures and whole cultures. In cell-free cultures, cell-free enzymes and sterilized cultures, no living cells were existed. Except cells, the adding volume of all liquid cultures was 1 mL. Cells were harvested from 10 mL broth, and added into *C. acetobutylicum* TSH1 directly.

Table 1 shows cell growth and solvent production under both anaerobic and microaerobic conditions by re-constructed symbiotic systems. Under anaerobic conditions, cells grew well and solvents (ABE) were produced for all the cases. However, under microaerobic conditions, solvents were produced only when living *B. cereus* TSH2 cells were supplied, i.e. with cells or whole cultures addition. The results indicated that living cells of *B. cereus* TSH2 offered *C. acetobutylicum* TSH1 to grow and produce butanol under microaerobic conditions, but the products and enzymes of *B. cereus* TSH2 didn’t have this ability. So, it was the *B. cereus* TSH2 cells protecting *C. acetobutylicum* TSH1 from environmental influences, but not the products or components of *B. cereus* TSH2. As an aerobic bacteria, *B. cereus* TSH2 could consume the residual oxygen and sustain an anaerobic environment. Dead cells or parts of cell lost the ability to consume oxygen and failed to rescue *C. acetobutylicum* TSH1 under microaerobic conditions.

**Table 1:** Cell growth and solvents (ABE) production by re-constructed systems

Re-constructed systems		Aerobic conditions		Anaerobic conditions	
*C. acetobutylicum* cultures	*B. cereus* TSH2 cultures	Cell growth [21]	ABE (g/L)	Cell growth	ABE (g/L)
*C. acetobutylicum* TSH1	Cells ^a^	+	13.9 ± 0.9	+	15.6 ± 0.4
	Cell-free cultures^b^	−	−	+	16.0 ± 0.8
	Cell-free enzymes^c^	−	−	+	16.6 ± 1.2
	Sterilized cultures^d^	−	−	+	15.1 ± 0.3
	Whole cultures^e^	+	16.5 ± 1.2	+	16.8 ± 0.5

^a^Cells were harvested from 10 mL broth by centrifugation at 10,625*g* for 5 min

^b^Cultures were filtered using a 0.22 μm pore size filter

^c^Enzymes were obtained by cells subjected to ultrasonic treatment at 200 W on ice for 10 min (10 s interval every 30 s), and filtered with a 0.22 μm pore size filter

^d^Cultures were obtained by the whole culture of *B. cereus* TSH2 autoclaved at 121 °C for 20 min

^e^Cultures were the fresh broth of *B. cereus* TSH2

### Dynamic dissolved oxygen variation during fermentation process

**Fig. 2:**
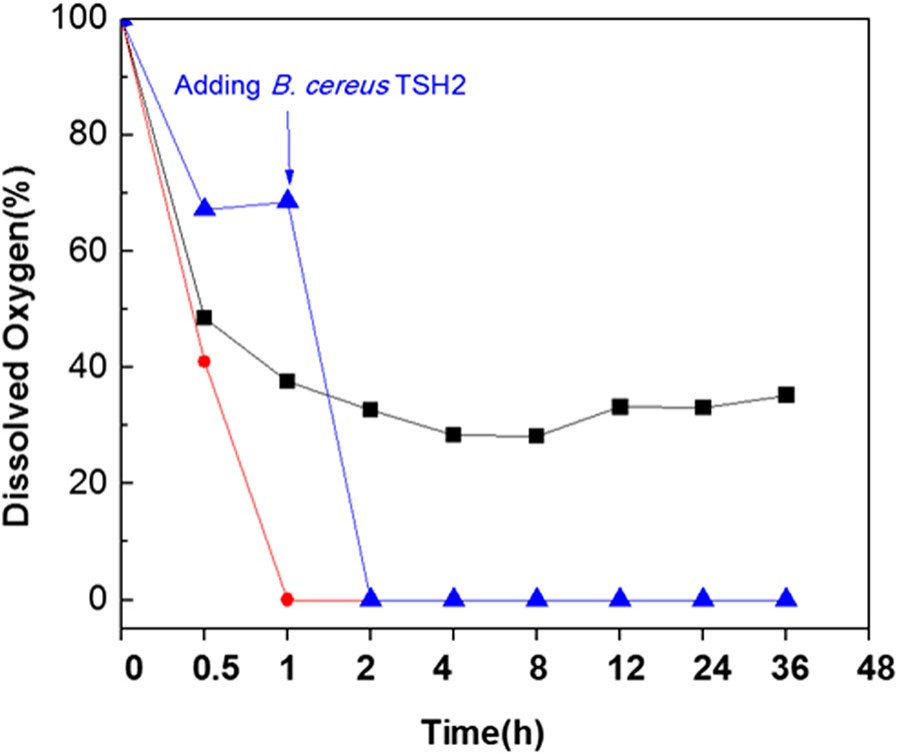
Time courses of dissolved O_2_ concentrations in different cultures. *Filled square* *C. acetobutylicum* TSH1, *filled circle* *C. acetobutylicum* TSH1+ *B. cereus* TSH2/0 h (adding *B. cereus* TSH2 at the beginning), *filled triangle* *C. acetobutylicum* TSH1+ *B. cereus* TSH2/1 h (adding *B. cereus* TSH2 at 1 h). The experiment was performed in 5 L bioreactor at 37 °C, the seed of *C. acetobutylicum* TSH1 was from a culture of P_2_ medium under 37 °C for 48 h, the seed of *B. cereus* TSH2 was from a culture of LB medium under 37 °C for 24 h

Dynamic dissolved oxygen variation of symbiotic system during fermentation process was studied in 5 L bioreactor. The symbiotic system was re-constructed by co-culture of *C. acetobutylicum* TSH1 and *B. cereus* TSH2, with inoculum ratio of 5 and 0.5 % respectively. A single culture of *C. acetobutylicum* TSH1 was carried out in the same bioreactor as control. The results showed in Fig. 2. For the single culture of *C. acetobutylicum* TSH1, dissolved oxygen decreased gradually after inoculating and kept at 33.1 % after 24 h. In contrast, dissolved oxygen was exhausted quickly within 1 h in the symbiotic system. Further study was carried out in a system in which *C. acetobutylicum* TSH1 was inoculated at the beginning and then *B. cereus* TSH2 was inoculated after 1 h of the fermentation. Under such condition, dissolved oxygen decreased to 76.3 % and kept at this level, however, it decreased sharply to zero in 1 h after *B. cereus* TSH2 addition. The results demonstrated clearly that *B. cereus* TSH2 consumed dissolved oxygen in the culture and offered anaerobic condition for *C. acetobutylicum* TSH1, thus oxygen tolerance of the symbiotic system was guaranteed by *B. cereus* TSH2 addition.

*Clostridiums* are commonly classified as strict anaerobes, and oxygen is toxic or lethal to these bacteria. However, some of *Clostridiums* showed tolerance to oxygen and could survive for a short time when exposed to air [23, 24]. It has been reported that an oxygen scavenging system existed in *C. acetobutylicum*, which included many proteins, such as NADH oxidase, superoxide reductase (SOR), NADH:rubredoxin oxidoreductase (NROR), rubredoxin (Rd), flavoprotein A2 (FprA2), desulfoferrodoxin (Dsr), and rubperoxin (Rpr) [25–27]. These proteins constituted an alternative detoxification system for *C. acetobutylicum* to survive in short period of aeration. To scavenge oxygen,this alternative pathway used the ‘reducing power’ (NADH and NADPH) in cell as substrate. If cell activity was high and ‘reducing power’ was sufficient, anaerobic conditions would be established, and cell growth and metabolism would be started on [8]. Unfortunately, *C. acetobutylicum* TSH1 usually failed to build anaerobic conditions for itself. Adding aerobic *B. cereus* TSH2 to medium would help *C. acetobutylicum* TSH1 to consume residual oxygen and set up anaerobic condition (Fig. 2). The symbiotic system constructed by co-culture of *C. acetobutylicum* TSH1 and *B. cereus* TSH2 does not require addition of any costly reducing agent or flushing with N_2_ to ensure anaerobic conditions, which makes ABE fermentation more economy.

### Effect of inoculation ratio of *C. acetobutylicum* TSH1 and *B. cereus* TSH2 on butanol production

In symbiotic system, *B. cereus* TSH2 acted as an oxygen scavenger and maintained anaerobic condition for *C. acetobutylicum* TSH1. However, *B. cereus* TSH2 didn’t produce butanol in the fermentation process. To study the effects of *B. cereus* TSH2 on butanol production, fermentation with different inoculation ratios were performed in 100 mL shake flasks. *C. acetobutylicum* TSH1 inoculation ratio of 5 % was fixed and *B. cereus* TSH2 inoculation ratio was changed from 0 to 5 %.

As shown in Table 2, the concentration, yield and productivity of butanol were decreased with inoculation ratio of *B. cereus* TSH2 increasing. When the inoculation ratio of *B. cereus* TSH2 was 0.5 %, butanol concentration reached 11.9 g/L. At *B. cereus* TSH2 ratio of 5 %, only 9.1 g/L butanol was produced. The butanol yield and productivity also decreased from 0.23 g/g to 0.20 g/g and 0.16 g/L h to 0.11 g/L h, respectively. The possible reason was attributed to nutrients competition and products inhibition which was produced by *B. cereus* TSH2. Though *B. cereus* TSH2 offered the condition for *C. acetobutylicum* TSH1 cell growth and butanol production under microaerobic conditions, excessive presence of *B. cereus* TSH2 would be disadvantage to butanol fermentation. The optimized inoculation ratio of *B. cereus* TSH2 was 0.5 %, which was adopted in the following studies.

**Table 2:** Butanol concentration, yield and productivity with different *B. cereus* TSH2 inoculum ratios

Inoculum ratio		Butanol concentration (g/L)	Butanol yield (g/g)	Butanol productivity (g/L h)
*C. acetobutylicum* TSH1 (%)	*B. cereus* TSH2 (%)			
5	0	–	–	–
	0.5	11.9 ± 0.4	0.23 ± 0.01	0.16 ± 0.01
	1	11.3 ± 0.9	0.23 ± 0.01	0.15 ± 0.01
	2	10.1 ± 0.7	0.22 ± 0.01	0.15 ± 0.01
	5	9.1 ± 0.5	0.20 ± 0.02	0.11 ± 0.01

Unlike *B. cereus* TSH2, the role of *C. acetobutylicum* TSH1 in symbiotic system was butanol production. Therefore, butanol producing ability of the symbiotic system depended on vitality of *C. acetobutylicum* TSH1. Table 3 showed butanol production with different *C. acetobutylicum* TSH1 inoculation ratios. With inoculation ratio increased from 1 to 7 %, there was no significantly change on butanol concentration and yield, but butanol productivity was increased from 0.13 g/L h to 0.18 g/L h. Considering the butanol concentration, yield and productivity, the optimized inoculation ratio of *C. acetobutylicum* TSH1 was 5 %. Under this inoculation ratio, butanol concentration, yield and productivity reached 11.9 g/L, 0.23 g/g and 0.16 g/L h. The lag phase period was shortened and fermentation process was accelerated with the *C. acetobutylicum* TSH1 inoculation ratio increasing. For example, the fermentation was ceased at 88, 80, 72 and 64 h when the inoculation is 1, 3, 5 and 7 %, respectively. Because *C. acetobutylicum* TSH1 still was the main contributor for butanol production in the symbiotic system, the similar butanol concentrations and yields were obtained with different inoculation ratios. Based on the above results, the optimized inoculation ratio of 0.5 % *B. cereus* TSH2 and 5 % *C. acetobutylicum* TSH1 was adopted.

**Table 3:** Butanol concentration, yield and productivity with different *C. acetobutylicum* TSH1 inoculum volume

Inoculum volume		Butanol concentration (g/L)	Butanol yield (g/g)	Butanol productivity (g/L h)
*B. cereus* TSH2 (%)	*C. acetobutylicum* TSH1 (%)			
0.5	0	–	–	–
	1	11.2 ± 0.7	0.22 ± 0.01	0.13 ± 0.01
	3	11.8 + 0.6	0.23 ± 0.01	0.15 ± 0.01
	5	11.9 ± 0.8	0.23 ± 0.01	0.16 ± 0.01
	7	11.5 ± 0.5	0.22 ± 0.01	0.18 ± 0.01

### Batch fermentation of symbiotic system under microaerobic conditions

Based on the above results, batch fermentation of symbiotic system under microaerobic conditions was studied with 3 L P_2_ broth in 5 L bioreactor. For symbiotic system, the inoculation ratio of *C. acetobutylicum* TSH1 and *B. cereus* TSH2 was 5 and 0.5 %, respectively. Fermentation was performed under microaerobic conditions including two treatment. One was static culture without any anaerobic pretreatment and another was the culture with air sparging at a rate of 0.15 L/min (0.05 vvm). In addition, single culture of *C. acetobutylicum* TSH1 with and without anaerobic pretreatment (N_2_ flushing) were introduced as control.

**Fig. 3:**
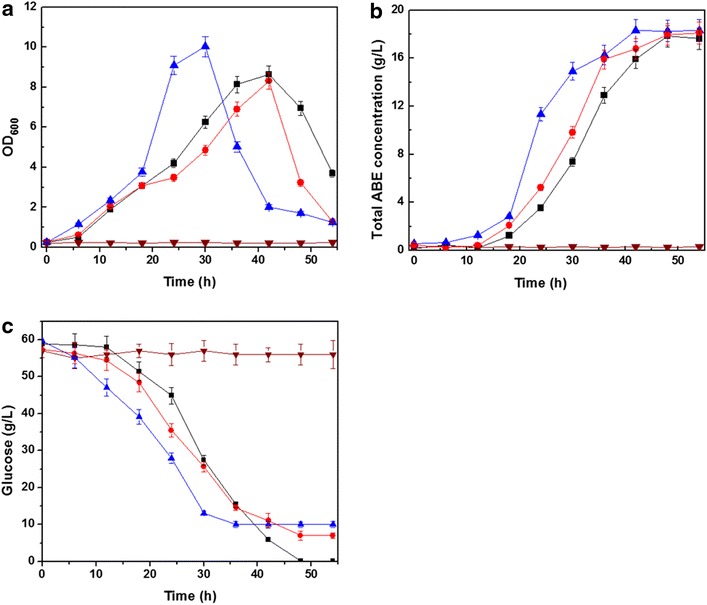
Biomass, ABE solvents, glucose concentration by symbiotic system and single culture of *C. acetobutylicum* TSH1. **a** Time courses of biomass (OD_600_), **b** Time courses of ABE solvents concentration, **c** Time courses of glucose consumption. *Filled triangle* *C. acetobutylicum* TSH1 with anaerobic treatment, *filled inverted triangle* *C. acetobutylicum* TSH1 without anaerobic treatment, *filled square* symbiotic system with 0.15 L/min (0.05 vvm) air flushing *filled circle* symbiotic system without anaerobic treatment

*C. acetobutylicum* TSH1 didn’t grow in P_2_ medium without anaerobic pretreatment. OD_600_ was still below 0.3 after 30 h (Fig. 3a). With anaerobic pretreatment by N_2_ flushing, *C. acetobutylicum* TSH1 grew well and OD_600_ reached 10.03 at 30 h. At 42 h, 18.3 g/L ABE was produced and 49.5 g/L glucose was consumed (Fig. 3a, b). The results proved that *C. acetobutylicum* TSH1 was obligate anaerobic bacteria. It was unable to grow and produce butanol under microaerobic conditions. In contrast, 18.1 g/L ABE was produced and 50.4 g/L glucose was consumed by the symbiotic system under static culture without anaerobic pretreatment. Furthermore, cells grew well and produced 17.8 g/L ABE in symbiotic system with air sparging. Though some papers have reported that aerating a small amounts of air into the fermentation broth didn’t affect the cell growth of *Clostridium*, these researches either used periodic aeration to control ORP (oxidoreduction potential) level or aerated with 5 % O_2_/95 % N_2_ mixed gas [28, 29]. In this study, even when air was continuously aerated into bioreactor, the symbiotic system still produced ABE solvents (Fig. 3b). To our knowledge, this is the first report about butanol production by a symbiotic system with continuous air sparging. The results also confirmed that oxygen tolerance of symbiotic system was much higher than that of the single culture. Compared with the condition of anaerobic pretreatment, fermentation rate of symbiotic system were slower. The fermentation of *C. acetobutylicum* TSH1 with nitrogen pretreatment finished at 42 h. And the fermentation of with symbiotic system air sparging and without nitrogen pretreatment finished at 48 h.

Product titers, yields, and productivities were shown in Table 4. Butanol titers obtained in these cultures were similar, but yields and productivities were slightly different. For *C. acetobutylicum* TSH1, about 11.1 g/L butanol was obtained, and for the symbiotic system, 11.0 g/L and 11.2 g/L butanol was obtained, respectively. Butanol yield of *C. acetobutylicum* TSH1 was 0.22 g/g, which was similar with artificial symbiotic system without anaerobic pretreatment. But yield of symbiotic system with air flushing was 0.18 g/g. Compared with the nitrogen pretreatment and static culture, continuous air flushing could take away a little solvent from the broth, so the solvent loss rate was higher. And another possible reason is excessive oxygen is disadvantage to the butanol production under microaerobic condition. Butanol productivity of *C. acetobutylicum* TSH1 with anaerobic pretreatment was 0.26 g/L h. In contrast, productivities of symbiotic system without anaerobic pretreatment and with air flushing were 0.21 and 0.23 g/L h, respectively. This phenomenon was corresponding to the slower cell growth and glucose consumption in symbiotic system cultures (Fig. 3). The slower fermentation rate of symbiotic system cultures could be attributed to inhibition of oxygen, as both of the cultures were carried out without anaerobic pretreatment. These results were agreed with the observations of Dwidar M et al. [30] and indicated that co-culture of *C.acetobutylicum* and *Bacillus* produced butyric acid after a longer lag time compared with nitrogen purging pretreatment.

**Table 4:** Comparison of batch ABE fermentations by *C. acetobutylicum* TSH1 and symbiotic system

	*C. acetobutylicum* TSH1 with pretreatment	Symbiotic system without pretreatment	Symbiotic system with air sparging
Glucose consumed (g/L)	49.5 ± 1.0	50.4 ± 1.8	58.6 ± 1.0
Acetone (g/L)	4.9 ± 0.1	4.9 ± 0.1	3.1 ± 0.2
Butanol (g/L)	11.1 ± 0.9	11.0 ± 0.5	11.2 ± 0.7
Ethanol (g/L)	2.3 ± 0.04	2.2 ± 0.1	2.6 ± 0.07
Total ABE (g/L)	18.3 ± 1.8	18.1 ± 1.2	16.9 ± 0.9
Butanol yield (g/g)	0.22 ± 0.01	0.22 ± 0.01	0.18 ± 0.01
ABE yield (g/g)	0.37 ± 0.01	0.36 ± 0.01	0.29 ± 0.01
Butanol productivity (g/L h)	0.26 ± 0.01	0.21 ± 0.01	0.23 ± 0.01
ABE productivity (g/L h)	0.44 ± 0.01	0.38 ± 0.01	0.37 ± 0.01

### Dynamics of abundance of *C. acetobutylicum* TSH1 and *B. cereus* TSH2 in the symbiotic system

Under microaerobic condition, *B. cereus* TSH2 provided anaerobic environment for *C. acetobutylicum* TSH1. However, the mutual mechanism of these two strains was not clear yet. In order to illustrate the interspecies relationship between *C. acetobutylicum* TSH1 and *B. cereus* TSH2, dynamic relative abundance during fermentation process under microaerobic static conditions were quantified by real-time PCR. The initial inoculation ratio of *C. acetobutylicum* TSH1/*B. cereus* TSH2 (C/B) was 10:1(5 % *C. acetobutylicum* TSH1: 0.5 % *B. cereus* TSH2), 1:1(2.5 % *C. acetobutylicum* TSH1: 2.5 % *B. cereus* TSH2) and 1:10 (5 % *C. acetobutylicum* TSH1: 0.5 *B. cereus* TSH2), respectively.

**Fig. 4:**
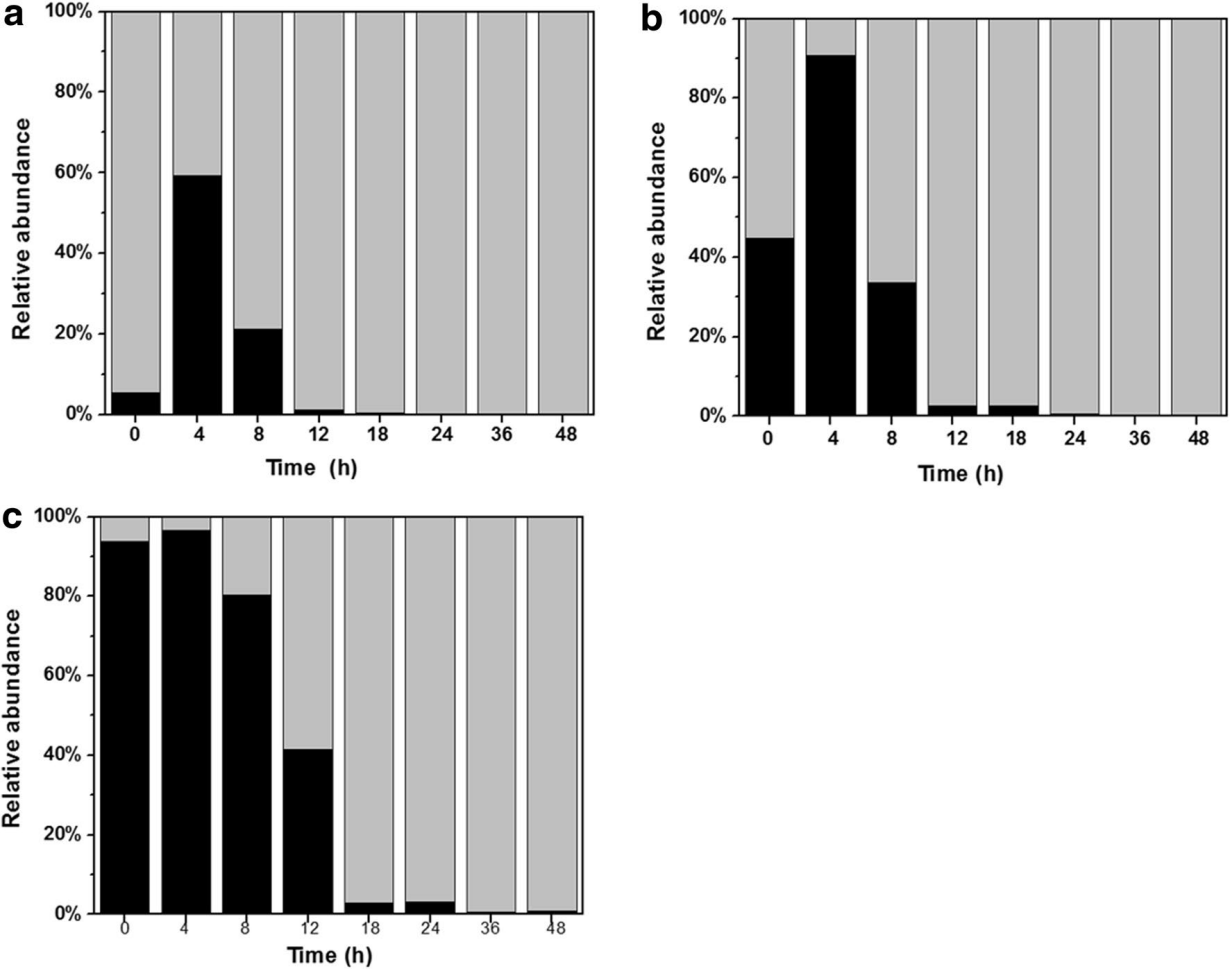
Dynamics of relative abundance for *C. acetobutylicum* TSH1 and *B. cereus* TSH2 at different inoculation ratios. **a** inoculation ratio of *C. acetobutylicum* TSH1/*B. cereus* TSH2 was 10:1, **b** inoculation ratio of *C. acetobutylicum* TSH1/*B. cereus* TSH2 was 1:1, **c** inoculation ratio of *C. acetobutylicum* TSH1/*B. cereus* TSH2 was 10:1. *Gray*, relative abundance of *C. acetobutylicum* TSH1, *Black*, relative abundance of *B. cereus* TSH2

When C/B ratio was 10:1, *C. acetobutylicum* TSH1 accounted for 94.5 % of the whole population at the beginning, whereas *B. cereus* TSH2 was only 5.5 % (Fig. 4a). However, the ratio of *B. cereus* TSH2 increased fast and reached 59.2 % at 4 h, while ratio *C. acetobutylicum* TSH1 declined to 40.2 %. Correspondingly, dissolved oxygen was totally exhausted in this period (Fig. 2). The abundance of *B. cereus* TSH2 started to decline sharply from 4 to 12 h, and maintained at a very low level of 0.15 % after 24 h. At the same time, abundance of *C. acetobutylicum* TSH1 increased to 99.85 % of the whole population. The high ratio of *Clostridium* and low ratio of *Bacillus* composition in the symbiotic system was beneficial for butanol production.

When C/B ratio was changed from 10:1 to 1:1 and 1:10, the abundance of *B. cereus* TSH2 increased and abundance of *C. acetobutylicum* TSH1 decreased correspondingly, especially in the initial 4 h after inoculation. The highest proportion of *B. cereus* TSH2 was 96.5 % when the C/B ratio was 1:10. However, the abundance of *B. cereus* TSH2 sharply decreased to 0.92 % in the later phase of fermentation which was similar to the case of C/B ratio at 10:1, and *C. acetobutylicum* TSH1 finally became dominant species in the symbiotic system. The difference between these two conditions was that the abundance decline rate of *B. cereus* TSH2 at C/B ratio 10:1 was lower than that of C/B ratio at 1:10. Higher proportion and longer declining period indicated that more nutrients were consumed by *B. cereus* TSH2. The results explained why excessive of *B. cereus* TSH2 in the symbiotic system had a negative effect on butanol production.

In general, as shown by qPCR, both *C. acetobutylicum* TSH1 and *B. cereus* TSH2 were coexisted during the fermentation process under microaerobic static conditions. At the beginning of fermentation, there was still a certain amount of dissolved oxygen in the fermenter, aerobic *B. cereus* TSH2 grew primarily and consumed oxygen. In this period, *B. cereus* TSH2 was an assistant for *C. acetobutylicum* TSH1. Once anaerobic condition established, *C. acetobutylicum* TSH1 grew quickly and became the dominant species. During the fermentation process, *C. acetobutylicum* TSH1 released large amounts of gas which prevented air into the fermenter and maintained anaerobic condition by itself. Furthermore, nutrient components of P_2_ medium and anaerobic condition were more favorable for *Clostridium*. So, *C. acetobutylicum* TSH1 became dominant species during fermentation process. The results of qPCR indicated that symbiotic system was able to reach a balance with high ratio of *Clostridium* and low ratio of *Bacillus* composition by natural cooperation and competition between the two strains. This was another advantage in using symbiotic system for ABE fermentation.

## Conclusions

Based on symbiotic system TSH06, a new symbiotic system was successfully re-constructed by co-culture of two different specified strains, anaerobic, solventogenic *C. acetobutylicum* TSH1 and aerobic, non-solventogenic *B. cereus* TSH2. Different from the traditional butanol fermentation strains, this symbiotic system showed higher oxygen tolerance and was able to produce butanol under microaerobic conditions. In 5 L bioreactor with 0.15 L/min (0.05 vvm) air sparging, 11.2 g/L butanol and 17.8 g/L ABE was obtained by the symbiotic system. This was the first report on butanol production by symbiotic system with continuous air sparging.

In this symbiotic system, *B. cereus* TSH2 was confirmed to be a good partner for *C. acetobutylicum* TSH1 by supplying an anaerobic environment. At the initial of fermentation, *B. cereus* TSH2 grew firstly and exhausted the residual oxygen in fermenter, then *C. acetobutylicum* TSH1 started to grow and produce solvents. Along with the fermentation, *C. acetobutylicum* TSH1 became dominant species and accounted for 99.85 % of the whole population in solventogenic phase. Increasing inoculation ratio of *B. cereus* TSH2 would affect butanol producing ability of the symbiotic system, but the relative abundance of each strain was not affected in the final broth, and *C. acetobutylicum* TSH1 was dominant species no matter how inoculation ratio was changed. The high ratio of *Clostridium* and low ratio of *Bacillus* composition during fermentation process indicated that this symbiotic system was an effective and easily controlled cultivation model for ABE fermentation under microaerobic conditions. It may be an attractive approach for butanol production.

## Methods

### Strains and medium

Both of *C. acetobutylicum* TSH1 and *B. cereus* TSH2 were isolated from TSH06, which was isolated from corn powder in the agricultural areas around the campus [21]. The stock culture was maintained in the form of a spore suspension in 25 % glycerol and frozen at −80 °C.

*C. acetobutylicum* TSH1 was anaerobically pre-cultured in a corn mash medium (6.5 % corn boiling at 100 °C for 30 min with constant stirring, and then autoclave at 121 °C for 20 min). It was incubated under static condition in an anaerobic chamber (Plus labs 855AC, US) at 37 °C for 18–24 h. The subculture was prepared in hemi-synthesis P_2_ medium containing: glucose (30 g/L), yeast extract(1 g/L), KH_2_PO_4_ (0.5 g/L), K_2_HPO_4_ (0.5 g/L), ammonium acetate (2.2 g/L), vitamins (1 mg/L para-amino-benzoic acid, 1 mg/L thiamin and 0.01 mg/L biotin), and mineral salts (0.2 g/L MgSO_4_•7H_2_O, 0.01 g/L MnSO_4_•7H_2_O, 0.01 g/L FeSO_4_•7H_2_O, 0.01 g/L NaCl), prepared according to the procedures described previously [31], incubated under static condition at 37 °C for 48 h. *B. cereus* TSH2 was aerobically pre-cultured in a Luria–Bertani (LB) medium, under shaking condition at 200 rpm and 37 °C for 12 h.

### Culture conditions

In the symbiotic system, inoculum volume of *C. acetobutylicum* TSH1 was 5 %, and *B. cereus* TSH2 was 0.5 % if not otherwise indicated. All of the inoculum size used volume ratio (vol %). To keep constant inoculation volume, the seed cultures were diluted to similar OD value before inoculation. Shake flask culture was carried out in 100 mL shaken flask with a rubber stopper, and working volume was 60 mL. The fermentation was performed statically at 37 °C without anaerobic treatment. Bioreactor fermentation was carried out in 5 L Biostat B plus fermenter (Sartorius company, Germany) containing 3 L P_2_ medium (55–60 g/L initial glucose) at 37 °C. Anaerobic fermentation was performed with N_2_ flushing pretreatment. Microaerobic fermentation was achieved by static culture or flushing with air at a rate of 0.15 L/min (0.05 vvm). Samples were taken at regular intervals to analyze biomass, substrate and solvents concentration.

### qPCR analysis

Genomic DNA was extracted according to the manufacturer’s instructions (TaKaRa MiniBest Bacterial Genome DNA Extraction Kit Ver. 3.0). The purity of DNA was checked by using a NanoDrop ND-2000 spectrophotometer (NanoDrop technologies, Wilmington, DE) and electrophoresis. Primers were designed according to 16S rRNA sequence difference between *B. cereus* and *C. acetobutylicum* listed in Table 5.

**Table 5:** Primers used for qPCR in this study

Primer	Sequence (5′–3′)	Targeted strain
B16SF	GTTGAATAAGCTGGCACC	*B. cereus* TSH2
B16SR	CGTGGGCTTTCACATCAGA	*B. cereus* TSH2
C16SF	GGGCTGCATTTCAAACTGGA	*C. acetobutylicum* TSH1
C16SR	GGGCTGCATTTCAAACTGGA	*C. acetobutylicum* TSH1

The qPCR was conducted using Bio-Rad CFX Connect Real-Time PCR Detection System (Bio-Rad Laboratories, Inc., Richmond, CA). The reaction mixture for each assay contained 12.5 μL of SYBR Premic EX Taq (TaKaRa Biotechnology Co., Dalian, China), 0.5 μL of each primer, and 1 μL (20 ng) of genomic DNA in a final volume of 25 μL. The PCR was performed using the following protocol: 95 °C for 2 min, following by 40 cycles at 95 °C for 10 s, and 55 °C for 30 s. After the PCR was over, a temperature gradient between 65 and 95 °C was performed for analysis of dissociation curves. The assay was performed at least three times for each sample.

To generate standard curves for quantification, two standard plasmids were used. Standard plasmid was constructed by pEASY-T1 vector ligation with PCR product of 16S rRNA, and then transformed to *E. coli* DH5α. Copy number was calculated by Eq. (1).

1 $$Copy \, number = \frac{DNA \, amount}{{DNA \, length \times 660 \times 1 \times 10^{9} }} \times 6.022 \times 10^{23}$$

qPCR reactions were run on serial dilutions of each standard plasmid to relate threshold cycle number(C_t_ value) to copy numbers of the target sequence and to generate standard curves for quantification in unknown samples. Typically, standard curves were linear across five orders of magnitude (10^7^–10^2^ copies, R^2^ = 1–0.98).

Since 16S DNA is expressed with 11 and 13 copies in the genomes of *C. acetobutylicum* and *B. cereus*, respectively, the abundance of each strain in the co-culture system was determined by Eqs. (2) and (3).

2 $$Abundance \, of \, C.\,acetobutylicum = \frac{16s \, cace \, copy \, number/11}{16s \, cace \, copy \, number/11 + 16s \, bcer \, copy \, number/13}$$

3 $$Abundance \, of \, B.\,cereus = \frac{16s \, bcer \, copy \, number/13}{16s \, cace \, copy \, number/11 + 16s \, bcer \, copy \, number/13}$$

## Analytical methods

Cell density was determined by measuring OD_600_ using a UV–visible spectrophotometer (UV-2802PC; Unico, Shanghai, China). During the fermentation period, 1.0 mL sample was taken on time and centrifuged at 10,625*g* for 5 min. The supernatant was used for ABE andglucose concentrations analyses. ABE were measured by gas chromatography (GC 2010, Shimadzu Co., Kyoto, Japan) equipped with a flame ionization detector (FID) and a glass column (KB-5MS,25 m × 0.53 mm × 1.00 μm, Kromat Corporration, US). The temperature of the detector and injector were maintained at 260 and 240 °C, respectively.

Glucose concentration was determined by high-pressure liquid chromatography (HPLC) analysis (LC-20AT, ShiShimadzu Co., Kyoto, Japan). A HPX-87 H column (300 × 7.8 mm) (Bio-Rad, USA) was used with a mobile phase of 5 mM sulfuric acid and a flow rate of 0.80 mL/min at 65 °C. The oxygen concentration in the medium was measured with an oxygen electrode (Hamilton OXYFERM VP 225, Bonaduz, Switzerland). Butanol concentration was calculated as butanol produced in g per L of broth. Butanol yield was calculated as g of butanol produced per g of sugar utilized and was expressed in g/g, butanol productivity was calculated as butanol produced in g per liter of broth divided by the fermentation time and was expressed in g/L h.
